# One-Step Encapsulation of Capsaicin into Chitosan–Oleic Acid Complex Particles: Evaluation of Encapsulation Ability and Stability

**DOI:** 10.3390/polym14112163

**Published:** 2022-05-26

**Authors:** Takashi Kuroiwa, Yoshiki Higuchi

**Affiliations:** 1 Department of Applied Chemistry, Faculty of Science and Engineering, Tokyo City University, Tokyo 158-8557, Japan; g1416076@tcu.ac.jp; 2 Advanced Research Laboratories, Tokyo City University, Tokyo 158-0082, Japan

**Keywords:** polyelectrolyte, molecular complexation, colloidal carrier, food dispersion, storage stability

## Abstract

Capsaicin (CAP) demonstrates a potential for application in the food and pharmaceutical industries owing to its various attractive health benefits, including anti-cancer, anti-inflammatory, and antioxidant activities. However, the application of CAP is often limited by its low solubility in water, low bioavailability, and strong pungency. In this study, a simple one-step method for the stable encapsulation and dispersion of CAP in aqueous media was developed using polyelectrolyte complex particles formed by chitosan (CHI) and oleic acid (OA). Homogeneous particles with mean diameters below 1 μm were successfully prepared via spontaneous molecular complexation by mixing an aqueous solution of CHI with an ethanolic solution of OA and CAP. CAP was incorporated into the hydrophobic domains of the CHI–OA complex particles through hydrophobic interactions between the alkyl chains of OA and CAP. The factors affecting CAP encapsulation were investigated, and a maximum encapsulation yield of approximately 100% was obtained. The CHI–OA–CAP complex particles could be stored for more than 3 months at room temperature (22–26 °C) without resulting in macroscopic phase separation or degradation of CAP. We believe that our findings provide a useful alternative encapsulation technique for CAP and contribute to expanding its practical application.

## 1. Introduction

Capsaicin (CAP: *trans*-8-methyl-*N*-vanyllyl-6-nonenamide) is a natural alkaloid obtained from fruits of the capsicum plant family, which are well known as hot peppers. CAP is not only consumed as an ingredient in spicy food but can also be used as a pharmaceutical supplement owing to its health benefits, which include anti-cancer, anti-inflammatory, anti-obesity, analgesic, cardino-protective, anti-microbial, and anti-oxidant effects [1,2,3,4,5]. As CAP is an extremely hydrophobic molecule (log *P* = 3.2), it has low solubility in water (140 mg/L at 25 °C) [6]; thus, its application is often limited owing to its low processing ability and low bioavailability, as well as its strong pungency and mucosal irritation properties. To overcome these limitations, the encapsulation of CAP into colloidal carriers has been actively studied in the last decade [3,4].

Various methods for encapsulating CAP into colloidal carriers have been reported, including the utilization of amphiphilic molecular aggregates such as micelles [7], microemulsions [8], liposomes [9,10,11], cubosomes [12], utilization of emulsion-based preparations [13,14,15,16], utilization of microcapsules prepared by coacervation [17,18,19,20,21], in situ polymerization [22], solvent evaporation [23,24], spray-chilling [5,25], gel-entrapment [26], and utilization of molecular complexation/inclusion [27,28,29,30,31,32,33,34,35,36]. Among these methods, molecular complexation/inclusion methods, which entail “bottom-up” self-assembly processes involving a facile formation procedure and low energy consumption, exhibit application advantages. However, further advancement of this technological field is still desired for the development of facile and highly efficient preparation methods for novel colloidal carriers.

Polyion complex particles [37] are prepared by the layer-by-layer polyelectrolyte self-assembly [38,39], and the complexation of positively and negatively charged polysaccharides [40,41] are widely used in the field of drug encapsulation and delivery. To our best knowledge, however, these techniques have never been applied to CAP encapsulation. Recently, polyion complex particles were prepared by simple and spontaneous complexation of cationic chitosan (CHI) and anionic oleic acid (OA) [42]. They can be used to encapsulate CAP. CHI is obtained by the deacetylation of chitin, which is a major component of marine crustaceans, and demonstrates unique physicochemical and physiological properties derived from the primary amino groups of the D-glucosamine residues of CHI. OA is an unsaturated fatty acid that is typically found in liquid triglycerides derived from plants. CHI and OA can form a complex at a certain pH range, wherein both CHI and OA are positively and negatively ionized, respectively (the p*K*_a_ values of CHI and OA are reported as ~6.5 [43] and ~4.7 [44,45], respectively). The CHI–OA complex, prepared through a facile “one-step” preparation procedure, results in homogeneous fine particles with mean diameters smaller than 1 μm; this can be attributed to the combination of (1) electrostatic interactions between the -NH_3_^+^ groups of protonated CHI and the -COO^−^ groups of the ionized form of OA and (2) hydrophobic interactions between the alkyl chains of OA [42]. The aggregated OA alkyl chains form hydrophobic domains inside the CHI–OA complex particles, which can encapsulate hydrophobic bioactive molecules [42]. Owing to these features, CHI–OA complex particles can efficiently and stably encapsulate CAP. A facile preparation method and the high stability of the CHI–OA complex particle would be advantageous for the utilization of these complex particles as carriers in practical food and pharmaceutical applications.

Thus, the objective of this study was to demonstrate the potential of CHI–OA complex particles as carrier materials for the stable encapsulation of CAP. The dispersibility, encapsulation efficiency, encapsulation capacity, and storage stability of the complex particles were evaluated in this study, and the results are presented in this paper. We believe that the findings obtained in this study will be valuable for the development of practical alternative methodologies for utilizing the beneficial health effects of CAP.

## 2. Materials and Methods

### 2.1. Chemicals

CHI (Chitosan 10^®^, degree of deacetylation = 85%, viscometric average molecular weight [46] = 150,000) was purchased from FUJIFILM Wako Pure Chemical Corporation (Osaka, Japan). All other chemical reagents, including OA, ethanol, and CAP, were obtained from FUJIFILM Wako Pure Chemical Corporation (Osaka, Japan). All reagents were used without any further purification. The water used in all the experiments was purified using a Direct-Q water purification system (Merck Millipore Corporation, Billerica, MA, USA) and demonstrated a resistivity of 18.2 MΩ cm.

### 2.2. Encapsulation of CAP into CHI–OA Complex Particles

Encapsulation of CAP into CHI–OA complex particles was achieved based on the previous methodology [42] with some modifications. CHI powder (1 g) was added to 120 mL of water containing 20 mL of 2.0 M acetic acid, and the solution was stirred to dissolve the added CHI. The pH was adjusted to 5.0 with 1.0 M NaOH, and the solution with a volume of 200 mL yielded a 5 g/L CHI solution. The CHI solution was filtered through a filter paper to eliminate any undissolved impurities before use. OA and CAP were dissolved in ethanol (99.5%) at various OA/CAP concentrations. Next, 20 mL of the CHI solution was added to a cylindrical 50 mL glass vial, followed by dropwise addition of 2 mL of the OA–CAP mixture while stirring at 400 rpm using a magnetic stirrer at room temperature (22–26 °C) for 18 h to form the CHI–OA–CAP complexes. The molar mixing ratio of OA/CHI was defined as the ratio of the amount of OA (mol) to that of glucosamine residues in CHI [42]. The obtained CHI–OA complex particle suspension was centrifuged at 2000 rpm for 10 min to eliminate the coarse particles from the sample.

### 2.3. Measurement of Particle Diameter

The mean diameter of the CHI–OA complex particles was measured using a laser diffraction particle size analyzer (SALD-200 V ER, Shimadzu Corporation, Kyoto, Japan). The samples were diluted with water to obtain suitable turbidities for the measurements. The results are presented as the mean values ± standard deviation calculated from a minimum of three independent experiments.

### 2.4. Determination of the Amount of CAP Encapsulated

An aliquot of the CHI–OA–CAP complex particle suspension was collected and diluted with methanol. The sample was centrifuged at 2100× *g* for 5 min (Micro Six MS-1, As One Corporation, Tokyo, Japan), followed by filtration using a syringe-connected membrane filter (0.45 µm pores; Dismic 13HP045AN, Advantec Toyo Kaisha, Ltd., Tokyo, Japan) for the removal of precipitate. The absorption spectra of the filtered solution were measured using an ultraviolet–visible (UV–Vis) spectrophotometer (UV-1800, Shimadzu Corporation, Kyoto, Japan). The CAP content was determined using the absorbance recorded at 280 nm by plotting the values on a standard curve preliminarily obtained using the amount of CAP dissolved in methanol. To determine the free CAP content in the matrix surrounding the CHI–OA–CAP complex particles, the matrix was separated from the suspension via centrifugal ultrafiltration at 10,000× *g* (Centrisart^®^ I, molecular weight cut-off = 300,000, Sartrius AG, Göttingen, Germany).

### 2.5. Fourier Transform Infrared Spectroscopic Analysis

The CHI–OA–CAP complex particle suspension (2 mL) was vigorously mixed with 2 mL of *n*-hexane for at least 5 min using a vortex mixer. The mixture was centrifuged at 2500 rpm for 10 min, and the upper *n*-hexane phase was collected. Then, the *n*-hexane was evaporated under ambient condition and the dried residue was used for Fourier transform infrared (FTIR) spectroscopic analysis. The FTIR spectra were obtained on a Shimadzu IRSpirit FTIR spectrophotometer exhibiting an attenuated total reflection (ATR) unit (Shimadzu Corporation, Kyoto, Japan).

### 2.6. Small-Angle X-ray Scattering Measurements

Synchrotron small-angle X-ray scattering (SAXS) measurements were performed using the SAXS optics and detector system installed at BL-6A at the Photon Factory of the High Energy Accelerator Research Organization (KEK) (Tsukuba, Japan). Details regarding the beamline and experimental conditions can be found elsewhere [42,47,48]. The background (water) and sample (CHI–OA or CHI–OA–CAP complex particle suspensions) scattering intensities were measured for 20 s at 25 °C. The data were processed using the SAngler software (Ver 2.1.58, High Energy Accelerator Research Organization (KEK), Tuskuba, Japan) [49]. Silver behenate was used as a standard specimen to calibrate the scattering angle 2*θ*. The length of the periodic structure in the sample, *d*, was calculated using Bragg’s equation, as follows:

*d* = *λ*/2 sin (2*θ*/2) (1)

where *λ* is the wavelength of the X-rays (1.5 Å).

### 2.7. Stability of CHI–OA–CAP Complex Particles 

Stability of the CHI–OA–CAP complex particles during storage in dark at room temperature (22–26 °C) was evaluated. The glass vials containing CHI–OA–CAP complex particles were covered with aluminum foil to avoid photodegradation of CAP [50]. The aliquots were withdrawn periodically and the particle diameter distribution and the CAP content of the sample were measured as described in Section 2.3 and Section 2.4, respectively.

## 3. Results and Discussion

### 3.1. Preparation of the CAP-Loaded CHI–OA Complex Particle Suspension

**Figure 1:**
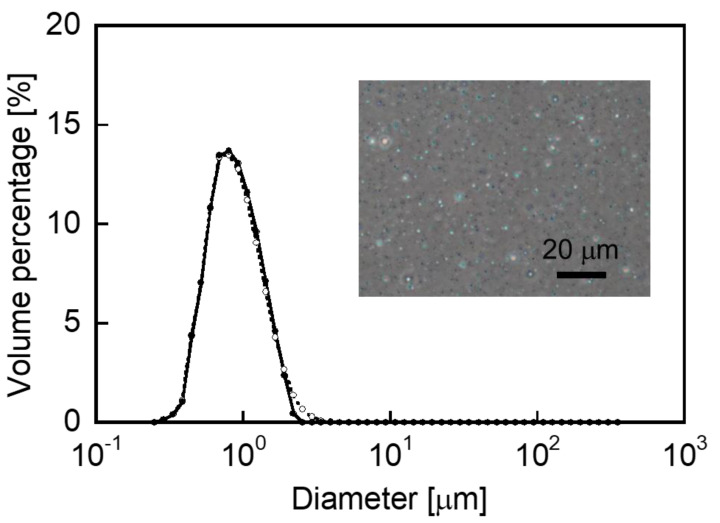
Particle size distribution of the chitosan−oleic acid (CHI−OA) complex particles in the presence (closed circles with solid line) and absence (open circles with dotted line) of capsaicin (CAP). The inset image shows the phase-contrast photomicrograph of the CHI−OA−CAP complex suspension.

Figure 1 illustrates the typical diameter distribution of the CHI–OA complex particle suspensions in the presence and absence of CAP, as well as a phase-contrast photomicrograph of the sample. A homogeneously turbid suspension was obtained by mixing the CHI solution with a mixture of OA and CAP in ethanol at room temperature for 18 h. In this experiment, 1.0 g/L of CAP was encapsulated into the CHI–OA complex particles. Mean particle diameters of approximately 0.8 μm were obtained for the samples prepared in the presence and absence of CAP. The diameter distributions for both samples were nearly identical; thus, it can be concluded that a mean diameter of <1 μm was maintained even after encapsulation of CAP into the particles. The CHI–OA complex particles thus demonstrate the advantage of a facile and highly reproducible preparation procedure.

### 3.2. Loading Characteristics of CAP

**Figure 2:**
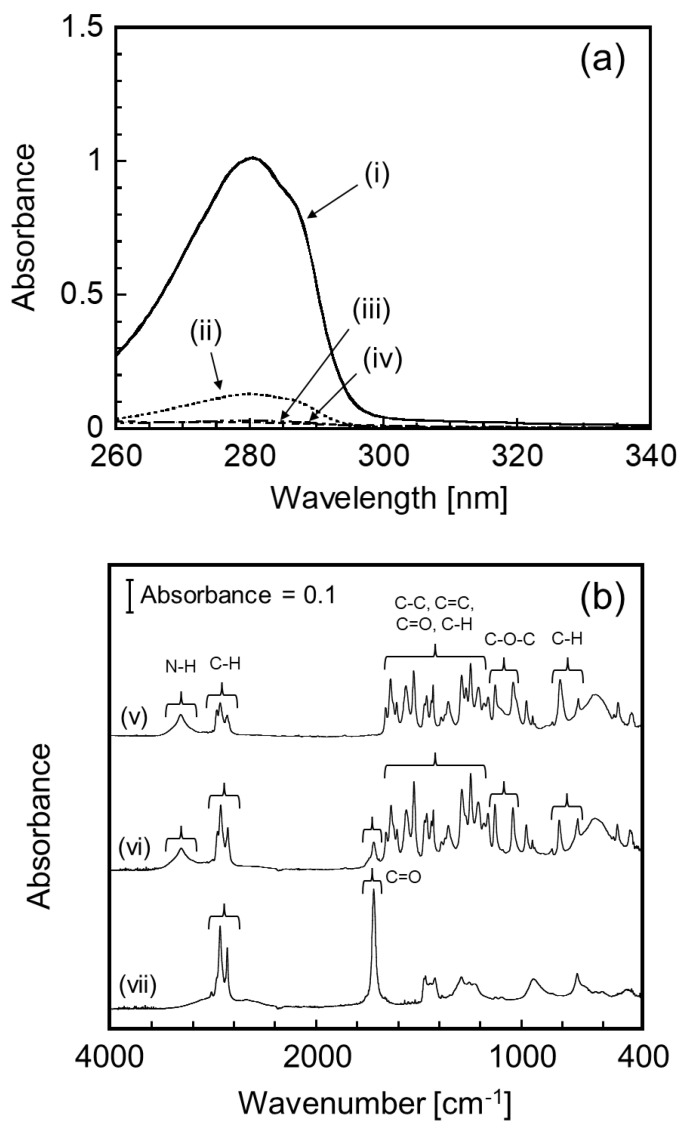
(**a**) Evaluation of capsaicin (CAP) loading via ultraviolet−visible (UV–Vis) spectroscopy: (**i**) chitosan (CHI)–oleic acid (OA)–CAP complex suspension (CAP concentration = 1.0 g/L), (**ii**) supersaturated dispersion medium (acetate buffer (0.2 M, pH 5.0): ethanol = 10:1), (**iii**) ultrafiltered permeate of the CHI–OA–CAP complex suspension (CAP concentration = 1.0 g/L), and (**iv**) CHI–OA complex suspension without the addition of CAP. (**b**) Fourier transform infrared (FTIR) spectra of (**v**) CAP, (**vi**) extract from the CHI–OA–CAP complex suspension, (**vii**) OA.

**Figure 3:**
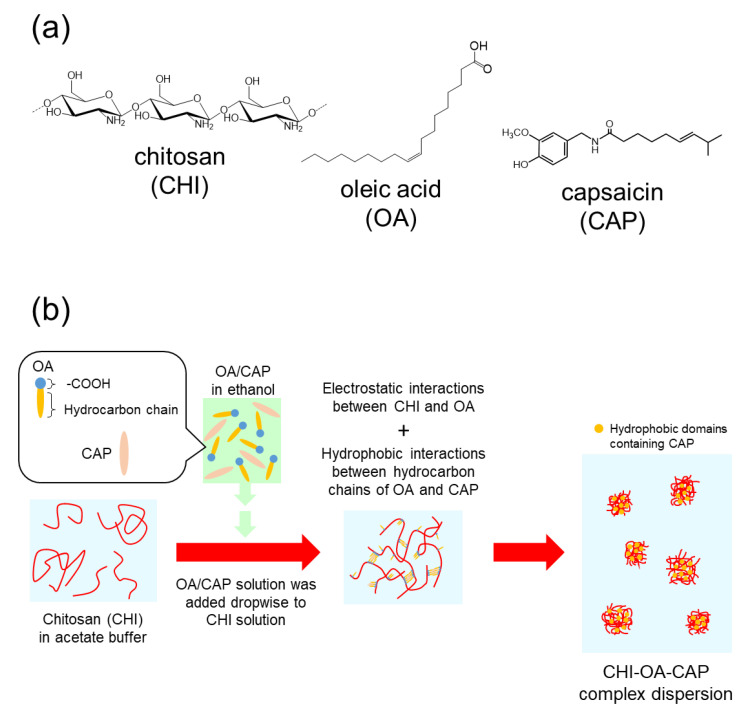
(**a**) Chemical structures of CHI, OA, and CAP and (**b**) schematic illustration of the possible encapsulation process of CAP into the CHI–OA complex particles.

CAP demonstrates a characteristic absorption peak at 280 nm, which allows evaluation of the CAP encapsulation into the CHI–OA complex particles via UV–Vis spectroscopy. Typical UV spectra for the various samples are shown in Figure 2a. Figure 2a(i) presents the spectrum of the CHI–OA complex particle suspension prepared using an initial CAP concentration of 1 g/L. The strong absorbance at 280 nm, which is significantly more intense than that of the dispersion medium (acetate buffer (0.2 M, pH 5.0): EtOH = 10:1) saturated with CAP (Figure 2a(ii)), confirms that CAP is successfully incorporated into the CHI–OA complex particle suspension. Interestingly, after removing the CHI–OA complex particles from the medium by centrifugal ultrafiltration, the medium contained a much smaller amount of CAP (Figure 2a(iii)) compared to its solubility in the medium, even though the total concentration of CAP in the sample suspension was much higher than that at its saturation level. It was also confirmed that the absorbance of the CHI–OA complex suspension without the addition of CAP was extremely low (Figure 2a(iv)). These results indicate that most of the CAP in the sample suspension was preferentially encapsulated by the CHI–OA complex particles. The hydrophobic domains formed by the aggregation of OA into the CHI–OA complex particles [42] led to the encapsulation of CAP via hydrophobic interactions between OA and CAP. This possible encapsulation process is depicted in Figure 3 with the chemical structures of CHI, OA, and CAP. It should be noted that more than 90% of CHI molecules in the sample were involved in the complex particle formation during this process [42].

In addition, CAP encapsulation was also confirmed by FTIR analysis. The absorption peaks in the spectrum of CAP (Figure 2b(v)) would be attributed to the following: N–H stretch; C–H stretch of CH_2_ and CH_3_; C–C stretch and C=C stretch in aromatic ring; C=O stretch; C–H bending of CH, CH_2_, CH_3_; C–O–C stretch; C–H bending in aromatic ring [51,52]. These characteristic peaks were also found in the extract from the CHI–OA–CAP sample (Figure 2b(vi)), as well as the peak attributed to the C=O stretch of COOH in OA (Figure 2b(vii)), which was extracted with CAP in the *n*-hexane phase during the extraction procedure (see Section 2.5). It should be noted that CHI was not extracted in the *n*-hexane phase because CHI was insoluble to *n*-hexane.

**Figure 4:**
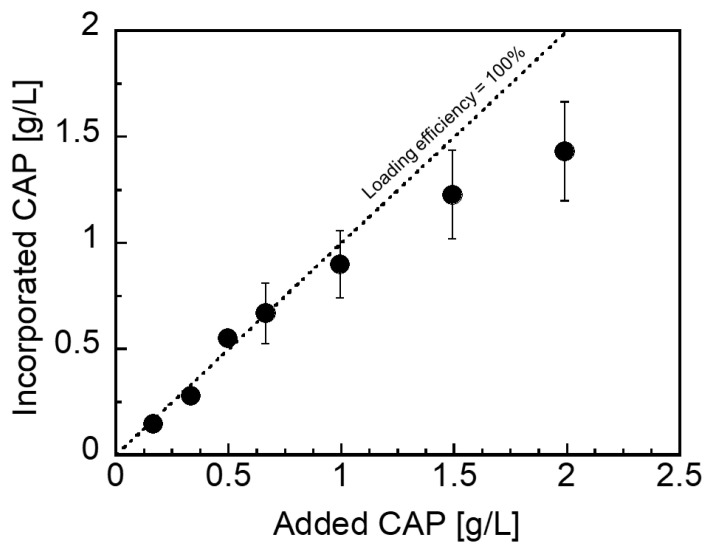
Effect of CAP concentration on the amount of CAP incorporated into the CHI–OA complex particle suspension. The dotted line represents a 100% CAP loading efficiency for the CHI–OA complex particles. The error bars indicate the standard deviations from the mean values determined from a minimum of three independent measurements.

CHI–OA–CAP complex particle suspensions containing various CAP concentrations were prepared to investigate the loading efficiency of the complex particles for CAP. The molar mixing ratio of OA to CHI was fixed at 0.2. Figure 4 illustrates the relationship between the amount of CAP added to and incorporated into the CHI–OA complex suspension. The amount of CAP incorporated into the complex particles increased linearly with an increase in the amount of CAP added, up to 1 g/L. Almost all the CAP added to the suspension was encapsulated by the complex particles with 100% efficiency (as indicated by the dotted line), even though an excess of CAP was added to the medium (~0.1 g/L). For CAP concentrations above 1 g/L, the CAP encapsulation efficiency was lower than 100%. At higher CAP concentrations, the hydrophobic domains in CHI–OA reached their maximum CAP incorporation capacities; therefore, a decrease in the encapsulation efficiency was observed.

**Figure 5:**
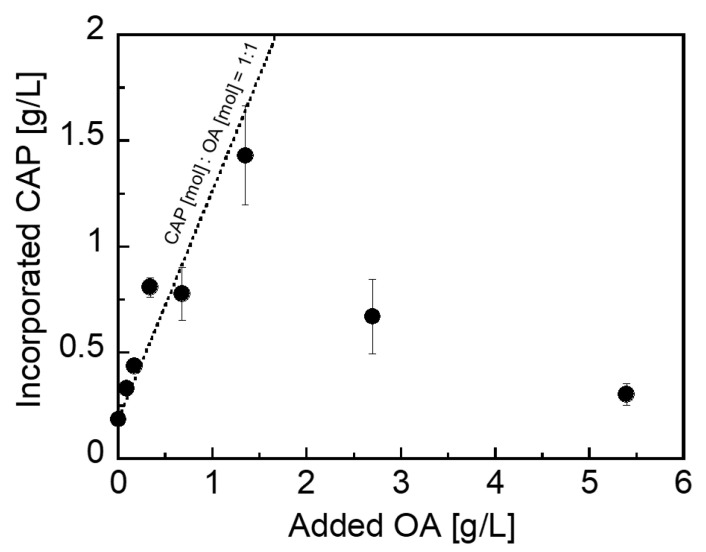
Effect of the OA concentration on the amount of CAP incorporated in the CHI–OA complex particle suspension. The dotted line represents a CAP:OA molar ratio of 1:1. The error bars indicate the standard deviations from the mean values determined by conducting a minimum of three independent measurements.

Next, we investigated the effect of the OA concentration on the capacity of CAP encapsulation. Since the initial amount of OA added during preparation of CHI–OA complex particles would affect the amount of the hydrophobic domains in the CHI–OA complex particles, the maximum capacity of CAP encapsulation into the CHI–OA complex particles would also be dominated by the added amount of OA. Here, the initial concentration of CAP was fixed at 2 g/L, which was an excess addition of CAP to its 100% encapsulation level. Figure 5 presents the relationship between the OA concentration and CAP incorporated into the CHI–OA complex particle suspension. At an OA concentration of less than 1.5 g/L, the amount of CAP incorporated increased linearly with an increase in the concentration of OA. This result suggests that the encapsulation capacity for CAP is dependent on the OA concentration in the complex particles. The dotted line in Figure 5 represents a molar ratio of incorporated CAP:OA of 1:1. This indicates that the OA hydrophobic domains are saturated with CAP at a molar ratio of CAP:OA of 1:1 when up to 1.5 g/L OA is added. The maximum encapsulation ability of the system could be predicted at this molar ratio as a function of the OA concentration. In contrast, the CAP encapsulation ability of the complex particles decreases at OA concentrations higher than 1.5 g/L. According to a previous study [42], CHI–OA complex particles prepared at a molar mixing ratio of OA to CHI higher than 0.4, which corresponds to ~2.7 g/L OA, tend to form large aggregates which are then separated by the centrifugation step performed after sample preparation. Therefore, the decrease in the amount of CAP incorporated could be attributed to a separation of the large CHI–OA–CAP aggregates, formed at a higher concentration of OA, by centrifugation of the prepared samples.

**Figure 6:**
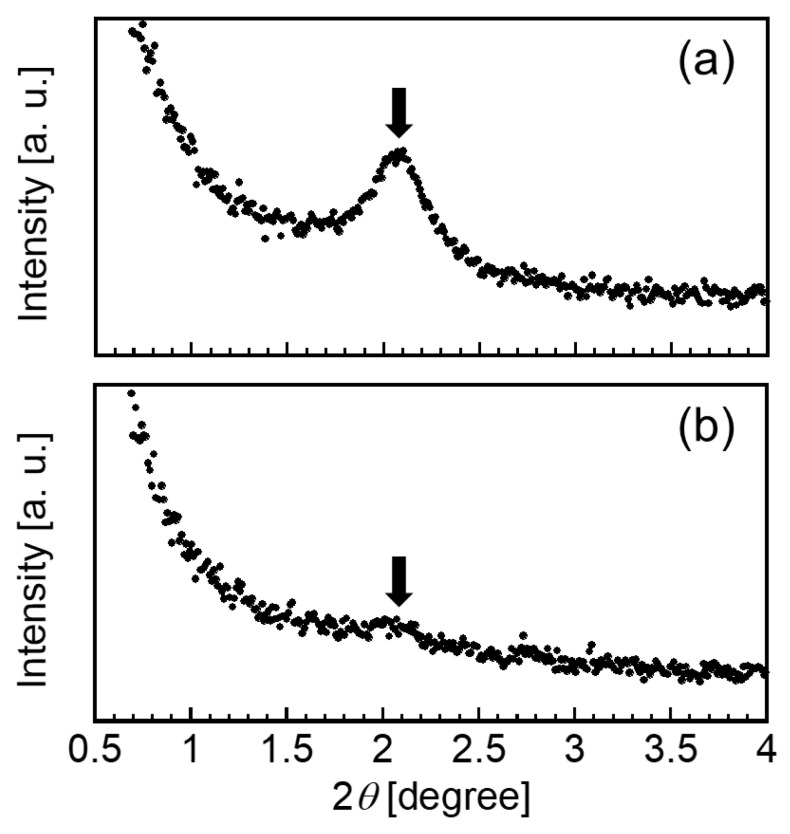
Small-angle X-ray scattering profiles of the CHI–OA complex particle suspension (**a**) in the absence and (**b**) presence of CAP prepared using an initial CAP concentration of 1.0 g/L at a molar mixing ratio of 0.2.

Based on the above discussion, we hypothesized that CAP was incorporated into the hydrophobic domains formed by the aggregation of hydrocarbon chains of OA in the CHI–OA complex particles. To investigate the loading mechanism of CAP in the complex particles, we conducted SAXS measurements. Figure 6 presents the SAXS profile of the CHI–OA complex particle suspension in the absence (Figure 6a) and presence (Figure 6b) of CAP. In the absence of CAP, a clear scattering peak is detected at 2*θ* = 2.1°, which corresponds to a characteristic periodic length, *d*, of 4.2 nm, as determined by Equation (1). This *d* value corresponds to the aggregation of OA molecules with ordered structures [42]. The incorporation of CAP into the CHI–OA complex particles at a CAP:OA ratio of 0.8 resulted in a decrease in the scattering peak intensity, indicating that the aggregated OA structure became less ordered. This result could be owing to the differences in the molecular shape and flexibility between CAP and OA. It would be difficult to maintain the ordered structure of OA when several CAP molecules are incorporated into the OA domains. In other words, the smaller scattering peak of CHI–OA–CAP indicates that CAP was incorporated into the aggregated OA domains by partitioning.

### 3.3. Evaluation of Stability

**Figure 7:**
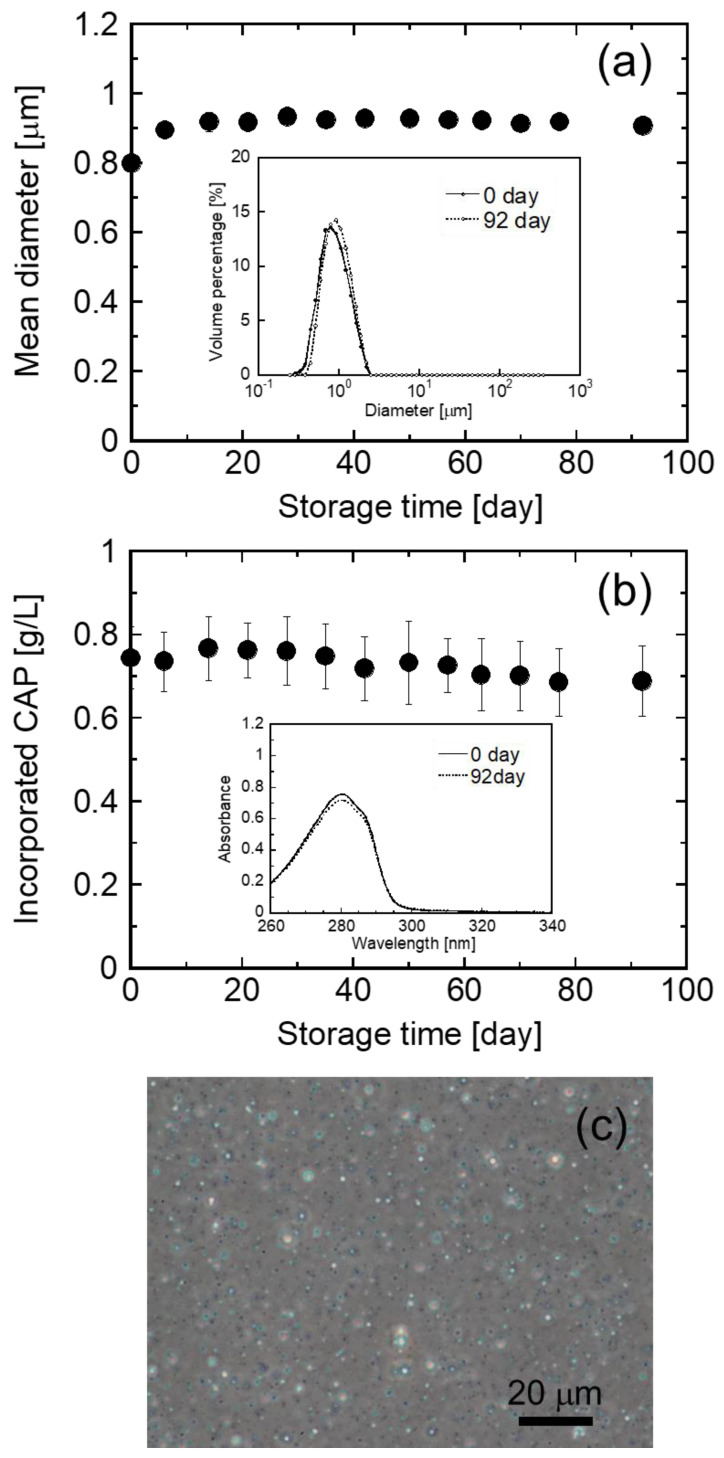
(**a**) Mean diameter of the CHI–OA–CAP complex particles and (**b**) CAP content in the CHI–OA–CAP complex particle suspension at room temperature over time. The inset graphs in (**a**,**b**) represent the diameter distribution of the CHI–OA–CAP complex particles and ultraviolet spectra of CAP extracted from the CHI–OA–CAP complex particles, respectively, after 0 and 92 days of storage. (**c**) Phase-contrast photomicrograph of the CHI–OA–CAP complex particle suspension stored for 92 days. The molar mixing ratio used was 0.2.

The stability of the dispersibility of the CHI–OA–CAP complex particles and encapsulated CAP was evaluated during storage in the dark at room temperature. Figure 7 presents the mean particle diameter and amount of incorporated CAP in the CHI–OA–CAP complex particle suspension, prepared at a molar mixing ratio of 0.2, as a function of time. The mean diameter of the CHI–OA–CAP complex particles and amount of CAP incorporated into the CHI–OA complex particles just after preparation (storage time = 0 day) were 0.81 μm and 0.74 g/L, respectively. Both the uniform particle diameter distribution and high CAP encapsulation efficiency were maintained over 3 months of storage without the formation of large aggregates or significant changes in the UV–Vis spectra owing to the oxidative degradation of CAP [53,54] (see the inset graphs in Figure 7a,b). In addition, there was no remarkable difference in the photomicrographs after 92-day storage (Figure 7c) from that of the initial sample (inset image in Figure 1), although a slight increase in the mean diameter of the complex particles was observed. These results reveal that CAP encapsulated into the CHI–OA complex particles can be stably stored for more than 3 months at room temperature. This feature of the CHI–OA–CAP complex particles is advantageous for applications in food and healthcare industries.

## 4. Conclusions

The factors influencing the encapsulation of CAP into CHI–OA complex particles were investigated. Consequently, the amount of encapsulated CAP in the CHI–OA complex particles was found to increase with an increase in the initial CAP concentration and the amount of OA present in the CHI–OA complex particles. The SAXS analysis results supported the proposed encapsulation mechanism of CAP into the CHI–OA complex particles, which stated that the CAP molecules were incorporated into the hydrophobic domains of the complex particles formed by the aggregation of hydrocarbon chains of OA via hydrophobic interactions. The stability of the prepared CHI–OA–CAP complex suspension during storage was examined at room temperature for over 3 months. The homogeneous dispersibility of the complex particles was successfully maintained at the sub-micrometer level, and the encapsulated CAP remained stable over the period investigated. We believe that the findings presented herein will contribute to the development of novel foods, beverages, pharmaceuticals, and other healthcare-related applications of not only CAP but also other hydrophobic bioactive molecules.

## Data Availability

Not applicable.
